# Kaempferide improves oxidative stress and inflammation by inhibiting the TLR4/IκBα/NF-κB pathway in obese mice

**DOI:** 10.22038/ijbms.2021.52690.11892

**Published:** 2021-04

**Authors:** Heng Tang, Qingfu Zeng, Nina Ren, Yunjie Wei, Quan He, Ming Chen, Peng Pu

**Affiliations:** 1 Department of Cardiology, The First Affiliated Hospital of Chongqing Medical University, Chongqing, PR China; 2 Department of vascular surgery, The Second Affiliated Hospital of Nanchang University; 3 Guangdong Online Hospital Clinic, Guangdong Second Provincial People’s Hospital, Guangzhou 510317, PR China; 4 Taihe Hospital Shiyan, Hubei, PR China

**Keywords:** Anti-inflammatory, Anti-oxidation, Kaempferide, Obesity, TLR4

## Abstract

**Objective(s):**

Kaempferide (Ka), a major natural active component of *Tagetes erecta* L, has numerous pharmacological effects such as anti-obesity, anticancer, and anti-hypertension. However, there is no clear evidence that Ka is directly related to inflammation and oxidative stress in obese mice. We aimed to explore the effects of Ka on inflammation and oxidative stress and its mechanism.

**Materials and Methods:**

The obese mice were induced by a high-fat diet (HFD). The anti-obesity effect was tested by liver and body weight, liver and adiposity index, and white adipose tissue. Blood sample analysis was used to detect the hypolipidemic and hypoglycemic effects. The anti-oxidation effect was assessed using GSH, SOD, MDA, CAT, T-AOC, and other indicators. The anti-inflammatory effect was assessed using TNF-α, MCP-1, and Adiponectin. Western blot and Real-Time PCR were used to evaluate the related signaling pathways.

**Results:**

Obesity, glycolipid metabolism disorder, inflammation, and oxidative stress developed in HFD mice. These changes can be effectively alleviated by Ka treatment for 16 weeks. Further studies have suggested that these beneficial effects of Ka may be associated with inhibition of the TLR4/IκBα/NF-κB signaling pathways.

**Conclusion:**

Ka possesses important anti-obesity, hypoglycemic, and hypolipidemic effects. The mechanism may be causally associated with the TLR4/IκBα/NF-κB signaling pathway, which improves inflammation and oxidative stress.

## Introduction

Obesity is closely related to coronary heart disease, atherosclerosis, hypertension, diabetes, and some tumors, and has become a serious problem that human beings have to face (1, 2). Relevant studies have shown that the liver, muscles, and fat of obese animals can produce excessive inflammation and oxidative stress (3, 4). The endocrine disorders, abnormal glycolipid metabolism, and energy imbalance caused by obesity will bring many adverse effects, which will seriously affect human health and even endanger life (5, 6).

Kaempferide (Ka), 3,5,7-trihydroxy-4′-methoxyflavone, 

the main active ingredient in *Tagetes erecta* L. has the effects of anti-obesity, anti-cancer, anti-hypertensive, and cardiovascular protection properties (7). Relevant studies have shown that Ka has a unique role in anti-oxidation and anti-inflammation (8, 9). Based on the above information, we speculate that these beneficial effects of Ka may be associated with the TLR4/IκBα/NF-κB pathway, which can improve obesity and glycolipid metabolism (10). However, no other research has confirmed this hypothesis.

Our study investigated the anti-inflammatory and anti-oxidant effects of Ka in HFD-induced obese mice and explored its target and the key pathway of anti-inflammatory and anti-oxidation. {He, 2019 #1}

## Materials and Methods

***Materials***

Ka (purity ≥92%) was purchased from Hubei ChuShengWei Chemistry Co. Ltd (Hubei, China). Commercial kits for triglyceride (TG) and total cholesterol (TC), high-density lipoprotein (HDL), and low-density lipoprotein (LDL) were purchased from Princeton Biotechnology Co., Ltd (Shanghai, China). T-AOC, GSH, GSH-Px, MDA, SOD, and CAT assay kits were obtained from Nanjing Jiancheng Bioengineering Institute Co. Ltd (Nanjing, China). Primers were synthesized by Sangon Biotechnology Co., Ltd (Shanghai, China). TLR4 signaling pathways related antibodies were obtained from Cell Signaling Technology.

***Animal experiments***

*Animal model*

The Animal Ethics Committee of the First Affiliated Hospital of Chongqing Medical University(CMU) approved this study. Male C57BL/6J mice (7 weeks old) were obtained from the CMU experimental animal center. The model was established according to our previously published research (11). Mice groups (4 groups in total, n=12 in each group): 

*ND* group: normal-diet-fed mice; 

*ND+Ka* group: normal-diet-fed mice treated with 10 mg/kg d^-1^ Ka; 

*HFD* group: high fat-diet-fed mice; 

*HFD+Ka* group: high fat-diet-fed mice treated with 10 mg/kg d^-1^ Ka.

The body weight was measured twice weekly.

*Sample preparation and collection*

The experiment ended at week 16. The mice were fasted overnight, weighed, and sacrificed (under anesthesia). Blood samples were collected and tissue samples, including liver and fat, were excised. The blood samples were centrifuged (4000 rpm, 4 °C, 30 min). Fatty liver index and adiposity index were calculated as follows: Fatty liver index [%] = liver weight [g]/body weight [g]×100; Adiposity index = white adipose tissue weight[g]/body weight[g]×100.

*Anti-oxidant activity in liver*

A certain amount of fresh liver samples was added into Tris–HCl (5 mmol/l containing 2 mmol/l EDTA, pH 7.4) and homogenized in a glass homogenizer. The samples were centrifuged at 1000 rpm and 4 °C for 10 min. The protein content in the supernatant was determined by the BCA method, and T-AOC, CAT, GSH, GSH-Px, MDA, and SOD of the supernatant were determined according to the instructions of the relevant kits.

*Real-time PCR*

TRIzol (Invitrogen) was used to extract total RNA from frozen pulverized mouse liver, then it was transcribed by a two-step method with Superscript First-Strand Synthesis System. SYBR Green PCR Master Mix (Applied Biosystems) was used to quantify the PCR products, and the results were normalized to β-actin gene expression. The primer sequences were listed in Table 1.

*Western blotting*

Liver tissue (n=4) was added into Radioimmunoprecipitation (RIPA) dissolution buffer and homogenized in a tissue homogenizer. The BCA kit was used to determine the protein concentration in the supernatant. Liver tissue lysate (50 μg) was used to carry out SDS polyacrylamide gel electrophoresis and transfer the protein to the FL membrane (microporous). The expression level of specific protein was standardized as GAPDH.

***Statistical analysis***

Data were expressed as mean±standard deviation (SD). Two-way analysis of variance (ANOVA) was used to analyze the significance of the differences among the groups, and then Tukey’s multiple comparison test was performed. *P*<0.05 was considered significant.

## Results

***Obesity and disorder of glycolipid metabolism was induced in HFD mice***

**Figure 1:**
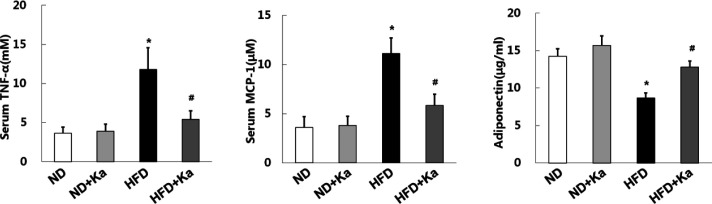
Kaempferide lowered the levels of inflammation factors in obese mice

The HFD mice developed obesity (Table 2), hyperlipidemia, hyperglycemia (Table 3), oxidative stress (Table 4), and inflammation (Table 5, Figure 1), which proved that the mouse model has been established successfully.

***Kaempferide alleviated obesity state***

The increased body weight indicated that HFD induced obesity successfully in mice (Table 2, *P*<0.05). Before sacrifice, the average body weight of the HFD group (38.2±1.11g) was significantly higher than that of the ND group (30.4±0.96 g) (*P*<0.01). But after Ka treatment, bodyweight, obesity index, liver weight, liver index, and white adipose tissue showed favorable changes (*P*<0.05, Table 2).

***The role of kaempferide in abnormal glycolipid metabolism***

The serum TC level of the HFD group (4.76±0.52 mM) was 140.4% higher than that of the ND group (1.98±0.35 mM) (*P*<0.01, Table 3), indicating that HFD mice had hyperlipidemia. After Ka treatment, TC level decreased significantly (*P*<0.05, Table 3). The changing trend of serum TG, LDL, and HDL was similar to that of serum TC (*P*<0.05, Table 3).

HFD induced a significant increase in blood glucose in mice (*P*<0.01). Ka treatment significantly reversed these changes (*P*<0.05, Table 3). 

***Kaempferide improves oxidative stress and inflammation***

*Kaempferoside alleviated oxidative stress state*

At the end of the experiment, levels of T-AOC, GSH-Px, CAT, GSH, and SOD decreased, while the MDA levels increased, indicating that the oxidative stress of HFD mice was increased. Ka treatment can effectively improve these changes (Table 4).

*Kaempferoside alleviated proinflammatory factor production*

TNF-α and MCP-1 are both pro-inflammatory factors, which can promote the occurrence of inflammatory reactions. In Figure 1, HFD results in increased serum TNF-α and MCP-1 levels in mice. However, these changes were reversed after the treatment of Ka.

In addition, HFD can reduce the level of adiponectin which is a target in an obesity-related inflammatory state. This change was reversed after Ka treatment (Figure 1).

*Molecular changes of inflammatory response-related genes*

NF-κB exists in almost all animal cells and plays a key role in the cellular inflammatory response. We evaluated the mRNA levels of NF-κB, IL-6, ICAM-1, VCAM-1, and TNF-α. In the HFD group, mRNA expressions of NF-κB, IL-6, ICAM-1, VCAM-1, and TNF-α increased by 255%, 246%, 271%, 212%, and 185%, respectively (*P*<0.05). These increased mRNA levels returned to near-normal levels after Ka treatment. (*P*<0.05).

*Changes in the expression of key proteins in the TLR4/IκB* *α* */* *NF* *-* *κB signaling pathway*

**Figure 2:**
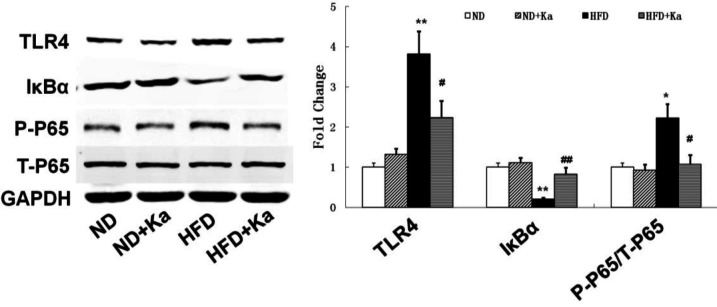
The possible molecular mechanisms of kaempferide in attenuating oxidative stress and inflammation in obese mice

A TLR4/IκBα/NF-κB signaling pathway is closely associated with inflammatory and oxidative stress. In Figure 2, HFD decreased the level of IκBα and increased the levels of TLR4 and P-P65. These changes returned to near normal level after Ka treatment. More evidence can be found in the semi-quantitative analysis (Figure 2).

**Table 1:** Sequences of the primers used in the PCR measurements

Gene	Sense	Sequence ( 5' to 3‘ )
TNF-α	TNF-α-FWD	CCGATGGGTTGTACCTTGTC
	TNF-α-REV	GGGCTGGGTAGAGAATGGAT
IL-6	IL-6-FWD	TCCTACCCCAATTTCCAATGC
	IL-6-REV	CATAACGCACTAGGTTTGCCG
ICAM-1	ICAM-1-FWD	TTCCGCTACCATCACCGTGT
	ICAM-1-REV	AGGTCCTTGCCTACTT
VCAM-1	VCAM-1-FWD	GGGAGACCTGTCACTGTCAACT
	VCAM-1-REV	GGACTTTATGCCCATTTCCTC
NF-κB	NF-κB-FWD	GCGAGAGAAGCACAGATACCA
	NF-κB-REV	GGTCAGCCTCATAGTAGCCA
β-actin	β-actin -FWD	CCACTGCCGCATCCTCTTCCTC
	β-actin -REV	TCCTGCTTGCTGATCCACATCT

Kaempferide exhibited a favorable anti-inflammatory effect. (A, B) Inflammatory factors (TNF-α,MCP-1) and (C) adipokines (adiponectin) changed markedly, after kaempferide treatment (n=6). All values are the mean±SEM. *P<0.01 vs ND, #*P*<0.01 vs HFD

HFD: high-fat diet

**Table 2:** Kaempferide decreased obesity and organ weights in C57 mice (n=12)

	ND	ND+Ka	HFD	HFD + Ka
Bodyweight(g)	30.4±0.96	30.7±1.02	38.2±1.11^**^	35.1±0.92^#^
Liver weight(g)	1.14±0.07	1.07±0.08	1.78±0.15^**^	1.30±0.09^#^
White adipose tissue(g)	1.11±0.13	1.14±0.16	3.26±0.52^**^	2.12±0.31^#^
Liver index(%)	3.75±0.24	3.49±0.26	4.66±0.28^*^	3.70±0.28^#^
Adiposity index(%)	3.65±0.26	3.71±0.33	8.27±0.68^**^	6.01±0.55^##^

Note: Ka-treated obese mice were compared with obese animals and with the controls. Ka lowered the body weights, organ weight, and index. Visceral fat includes epididymal fat pad, mesentery fat tissue, and abdominal adipose tissue. Adiposity Index = white adipose tissue weight(g) / body weight(g)×100. So was the liver index. All values are mean±SEM. *P<0.05, **P<0.01 vs ND, #*P*<0.05, ##*P*<0.01 vs HFD

HFD: high-fat diet; Ka: kaempferide; ND: normal-diet-fed

**Table 3:** Effects of kaempferide on glycolipid metabolism in C57 mice (n=12)

	ND	ND+Ka	HFD	HFD + Ka
Serum TC (mM)	1.98±0.35	2.13±0.41	4.76±0.52^**^	3.22±0.41^#^
Serum TG (mM)	0.67±0.08	0.53±0.06	1.22±0.24^*^	0.77±0.11^#^
Serum HDL (mM)	1.23±0.15	1.46±0.22	3.25±0.36^**^	2.17±0.24^##^
Serum LDL (mM)	0.52±0.05	0.39±0.07	1.16±0.11^**^	0.76±0.12^##^
Blood glucose(mmol/L)	5.58±0.64	5.67±0.63	8.63±1.02^*^	6.12±0.93^#^

Note: Ka treatment promoted a signiﬁcant decrease in serum glycolipid levels in comparison with obese animals. All values are mean±SEM. All values are mean±SEM. *P<0.05, **P<0.01 vs ND, #*P*<0.05, ##*P*<0.01 vs HFD

HFD: high-fat diet; Ka: kaempferide; ND: normal-diet-fed

**Table 4:** Effects of kaempferide on the activities of anti-oxidant enzymes and concentrations of non-enzymic anti-oxidants in the liver (n=12) of mice

	ND	ND+Ka	HFD	HFD + Ka
GSH(nmol/mg pro)	6.45±0.86	7.24±1.04	3.15±0.51^*^	5.66±0.69^#^
SOD(U/mg pro)	123.34±10.16	127.55±12.17	54.37±9.67^**^	92.64±11.45^#^
MDA(nmol/mg pro)	2.21±0.54	2.15±0.47	6.56±0.96^**^	3.84±0.82^#^
CAT(U/mg pro)	45.63±5.48	48.92±6.22	21.47±3.51^**^	37.24±4.92^#^
T-AOC(U/mg pro)	2.75±0.31	2.98±0.34	1.07±0.11^**^	2.26±0.25^##^
GSH-PX(U/mg pro)	718.47±59.35	764.29±48.36	348.53±26.13^**^	512.65±43.57^#^

Note: Kaempferide improved the antioxidative defense system as normal levels with increased activity of SOD, GSHpx, CAT, and the levels GSH. It also decreased the MDA content in the liver. All values are mean±SEM. *P<0.05, **P<0.01 vs SD, #*P*<0.05, ##*P*<0.01 vs HFD

SOD: superoxide dismutase; GSH-PX: glutathione peroxidase; CAT: catalase; MDA: malondialdehyde; HFD: high-fat diet

**Table 5:** Changes in hepatic expressions of inflammatory genes (n=6) of mice

	ND	ND+Ka	HFD	HFD + Ka
TNF-α	1.00±0.15	1.15±0.21	2.85±0.56^*^	1.79±0.31^#^
IL-6	1.00±0.13	1.08±0.16	3.46±0.87^*^	1.54±0.35^##^
ICAM-1	1.00±0.10	1.25±0.22	3.71±0.82^*^	2.18±0.53^#^
VCAM-1	1.00±0.12	0.91±0.20	3.12±0.64^*^	1.83±0.42^#^
NF-κB	1.00±0.11	1.03±0.21	3.55±0.72^*^	2.02±0.66^#^

Note: Expressions of inflammatory genes. Relative mRNA levels are expressed as a ratio relative to β-actin. All values are mean±SEM.*P<0.01 vs ND, #*P*<0.05, ##*P*<0.01 vs HFD

HFD: high-fat diet; Ka: kaempferide; ND: normal-diet-fed

(A) Representative images of Western blot analysis examining the expressions of TLR4/IκBα/NF-κB signaling pathway in each group; (B) Quantification of the expressions of the key proteins (n=4). GAPDH was used as an internal control. ND=normal diet group, ND+Ka=normal diet+Ka at 10mg/kg/day, HFD=high-fat diet, and HFD+Ka=high fat diet+Ka at 10mg/kg/day. Values are expressed as Mean±SEM. *P<0.05, **P<0.01 vs ND, #*P*<0.01, ##*P*<0.01 vs HFD

HFD: high-fat diet

## Discussion

This study demonstrated that Ka had promising effects of anti-inflammatory and anti-oxidation in obese mice at the dose of 10 mg kg^-1^ d^-1^, showing significant improvements in a range of parameters related to anti-obesity and improvement in glycolipid metabolism, such as body and liver weight, white adipose tissue, liver and adiposity index, TC, TG, LDL, HDL, and blood glucose. These protective effects of Ka might be closely associated with the TLR4/IκBα/NF-κB signaling pathway. In order to further explore and clarify the mechanism of these effects of Ka, we examined the status of oxidative stress and inflammation in obese mice and proved that Ka can improve oxidative stress and inflammation by inhibiting the TLR4/IκBα/NF-κB signaling pathway. 

An important aspect of this study is that Ka has beneficial anti-inflammatory effect in obese mice. It is well known that that obesity is currently defined as an inflammatory state and anti-inflammatory therapy is of great significance in the treatment of obesity (12, 13). TLR4 is a key receptor for both exogenous and endogenous ligand-induced inflammatory responses mediated by infectious stimuli and plays a key role in inflammatory response amplifiers (14). Activated TLR4 induces inflammatory responses and promotes the differentiation and maturation of antigen-specific acquired immune responses (15). NF-κB is an important nuclear transcription factor involved in inflammatory response, proliferation, and differentiation, located at the junction of the TLR4 downstream signaling pathway (16). At rest, NF-κB binds to the inhibitory protein IκB in the cytoplasm in an inactive form (17). When stimulated by extracellular signals, the IκB kinase (IKK) complex activates the IκB phosphorylation, and the free NF-κB rapidly moves to the nucleus and binds to specific κB sequences, inducing transcription of the gene involved (18, 19). Activation of NF-κB leads to the expression of ICAM-1, VCAM-1, and several inflammatory factors, leading to inflammatory responses and cell damage (20). Obesity increases the level of TLR4 protein in the liver, which induces degradation of IκB and dissociation of NF-κB (21). Ka therapy inhibited the activation of TLR4, promoted the expression of IκB, and further inhibited the expression of NF-κB, reduced the levels of ICAM-1, VCAM-1, and related inflammatory factors (MCP-1, IL-6, TNF-α), and alleviated the inflammatory damage of liver cells. This is consistent with the study of WuYajun *et al. *(22) on inflammatory injury of endothelial cells. 

Another interesting finding of this study is the anti-oxidation effect of Ka in obese mice. We speculate that this effect may also be affected by the TLR4/IκBα/NF-κB signaling pathway. MK Ko *et al*. (23) found that activation of TLR4 leads to oxidative stress damage to photoreceptor mitochondria. Wang *et al*. (24) also found that Picroside II protects the kidneys against oxidative stress through the TLR4/NF-κB pathway. Oxidative stress, a negative effect produced by free radicals in the body, is considered to be an important factor in aging and disease (25). In the process of metabolism, the endogenous anti-oxidant enzyme SOD can catalyze the superoxide radical degradation to hydrogen peroxide, and CAT catalyzes the decomposition of hydrogen peroxide into oxygen and water (26). As an important product of lipid oxidation, MDA is often used to reflect the degree of cell damage caused by oxidative stress (27). GSH, an important anti-oxidant and free radical scavenger in the human body can combine with toxic substances such as free radicals and heavy metals, and excrete them from the body (28). In this study, obese mice induced by HFD produced excessive oxidative stress, leading to a decrease in T-AOC. After Ka treatment, the MDA level decreased, and SOD activity, CAT, and GSH levels increased, indicating that Ka can protect and prevent oxidative stress injury. 

Although the anti-inflammatory and anti-oxidation effects of Ka have been preliminarily confirmed in some studies, most of these studies are about cancer and cardiovascular research. As far as we know, no other studies have directly demonstrated that the anti-inflammatory and anti-oxidation effects of Ka in obese mice were affected by the TLR4/IκBα/NF-κB signaling pathway. Our work provides the original evidence for Ka as a natural molecule with anti-inflammatory and anti-oxidation effects in obesity, highlighting its important underlying mechanism. However, the specific mechanism by which Ka regulates TLR4 *in vivo* is still not perfect. In addition, it is not clear whether the anti-inflammatory and anti-oxidation effects of Ka can promote each other. All these are worthy of further study.

## Conclusion

The results showed that the anti-inflammatory and anti-oxidation effects of Ka were closely related to the TLR4/IκBα/NF-κB signaling pathway, which could effectively improve obesity and glycolipid metabolism disorders in obesity. In conclusion, Ka may be a promising drug for the treatment of obesity and diabetes. 
